# High-Dose Buprenorphine Initiation in the Emergency Department Among Patients Using Fentanyl and Other Opioids

**DOI:** 10.1001/jamanetworkopen.2023.1572

**Published:** 2023-03-03

**Authors:** Hannah Snyder, Brendon Chau, Mariah M. Kalmin, Melissa Speener, Arianna Campbell, Aimee Moulin, Andrew A. Herring

**Affiliations:** 1 Department of Family and Community Medicine, University of California, San Francisco; 2 CA Bridge Program, Public Health Institute, Oakland, California; 3 Department of Biostatistics, Fielding School of Public Health, University of California, Los Angeles; 4 Department of Family Medicine, David Geffen School of Medicine, University of California, Los Angeles; 5 Department of Emergency Medicine, US Acute Care Solutions at Marshall Medical Center, Placerville, California; 6 Department of Emergency Medicine and Psychiatry, UC Davis Medical Center, Sacramento, California; 7 Department of Emergency Medicine and Internal Medicine, Highland Hospital–Alameda Health System, Oakland, California; 8 Department of Emergency Medicine, University of California, San Francisco

## Abstract

This cohort study examines buprenorphine treatment initiation, response, and follow-up among patients presenting to California emergency departments (EDs) who reported fentanyl or other opioid use.

## Introduction

CA Bridge is an implementation facilitation program for opioid use disorder (OUD) treatment in California emergency departments (EDs).^1^ CA Bridge guidelines include high-dose buprenorphine for most ED patients in withdrawal, with a starting dose of 8 to 16 mg.^2^ Patients and clinicians have raised concerns that individuals using fentanyl may have suboptimal responses to buprenorphine compared with individuals who use other opioids.^3,4,5^ Although fentanyl may be consumed unintentionally by patients using contaminated drugs, California also has a substantial market for fentanyl sold by name, often for use by smoking.^5^ During data collection, 64% of California opioid-involved overdose deaths involved fentanyl.^6^ In this cohort study, we compared buprenorphine treatment initiation, response, and follow-up treatment engagement between patients who did and did not report fentanyl use at CA Bridge EDs.

## Methods

We retrospectively abstracted data from electronic health records (EHRs) for patients with OUD who presented to 16 CA Bridge EDs from January 1 to April 30, 2020. Patients with OUD were included regardless of chief concern, current treatment, withdrawal, or treatment desires. The study followed Strengthening the Reporting of Observational Studies in Epidemiology (STROBE) reporting guidelines for observational research and was approved by the Public Health Institute institutional review board with a waiver of informed consent because the study posed minimal risk to participants, in accordance with 45 CFR §46. Site staff identified records, abstracted data using a standardized protocol, and conducted quality assurance procedures. Fentanyl use was defined by EHR documentation of patient report. If no fentanyl use was noted, the patient was coded as not using fentanyl; toxicology testing was not used. The primary outcome, follow-up engagement at 7 to 14 days (7-day follow-up) and 25 to 37 days (30-day follow-up), was defined by EHR documentation of buprenorphine or behavioral treatment through confirmation from the patient, outpatient practitioner, or the prescription drug monitoring program. For patients who did and did not report fentanyl use (independent variable), we estimated odds ratios and 95% CIs of outcomes, including buprenorphine administration or prescription and 7-day and 30-day follow-up, using a multivariable logistic linear mixed model controlling for a priori confounders: age, sex, race and ethnicity, housing status, methamphetamine use, and alcohol and benzodiazepine use. Race and ethnicity were included on the basis of prior evidence that members of racial and ethnic minoritized groups experience both limited access to buprenorphine treatment and reduced treatment engagement compared with White non-Hispanic patients. The correlation between patients within a given hospital was addressed with a random intercept. Statistical analysis was performed from March to August 2022 year using R statistical software version 4.0.3 (R Project for Statistical Computing).

## Results

There were 896 patients with OUD, of whom 87 (9.7%) reported fentanyl use. Their median (IQR) age was 35 (28-45) years, 613 (68.4%) were male, 60 were Black (6.7%), 229 (25.6%) were Hispanic, 412 (46.0%) were White, and 263 (29.4%) were unstably housed (Table 1). Seventy-five patients were excluded for incomplete data, and 6 were excluded for methadone use; 4 patients were excluded from the regression analysis alone (ie, other gender or disposition died or unknown). Hospitals were located in northern (9 hospitals), central (2 hospitals), and southern (5 hospitals) California; 4 hospitals (25%) were rural.

**Table 1.** Demographic Characteristics of CA Bridge Study Participants

Characteristic	Participants, No. (%)		
	Fentanyl use		Total (N = 896)
	Yes (n = 87)	No (n = 809)	
Age, median (IQR), y	29 (26-39)	36 (29-46)	35 (28-45)
Race and ethnicity^a^			
Black non-Hispanic	1 (1.1)	59 (7.3)	60 (6.7)
Hispanic	24 (27.6)	205 (25.3)	229 (25.6)
White non-Hispanic	44 (50.6)	368 (45.5)	412 (46.0)
Other or unknown^b^	18 (20.7)	177 (21.9)	195 (21.8)
Gender^a^			
Male	73 (83.9)	540 (66.7)	613 (68.4)
Female	14 (16.1)	268 (33.1)	282 (31.5)
Other or unknown	0	1 (0.1)	1 (0.1)
Housing status^a^			
Stable	53 (60.9)	452 (55.9)	505 (56.4)
Unstable	27 (31.0)	236 (29.2)	263 (29.4)
Other or unknown	7 (8.0)	121 (15.0)	128 (14.3)
Other substance use^c^			
Methamphetamine or stimulants	35 (40.2)	315 (38.9)	350 (39.1)
Alcohol or benzodiazepines	29 (33.3)	237 (29.3)	266 (29.7)
Heroin or pain medication	62 (71.3)	809 (100.0)	871 (97.2)
Prehospital management, emergency medical services engaged	21 (24.1)	136 (16.8)	157 (17.5)

^a^ Categories are mutually exclusive.

^b^ Includes American Indian or Alaskan Native, Asian, Native Hawaiian or other Pacific Islander, more than 1 race, and unknown or not reported.

^c^ Categories are not mutually exclusive.

Of the 492 patients (54.9%) who were administered buprenorphine, 44 (9.5%) used fentanyl. Overall, 439 patients (89.3%) initiated high-dose buprenorphine (8-32 mg) (Table 2). Follow-up at 30 days among patients administered buprenorphine was similar for those who did and did not report fentanyl use (36 patients [41.4%] vs 301 patients [37.2%]), vs 94 patients (23.3%) who were not administered buprenorphine. Among all patients who were administered buprenorphine, precipitated withdrawal was documented for 8 patients (1.6%). Among the subgroup of patients who reported fentanyl use, there were 2 cases (4.5%) of precipitated withdrawal. No precipitated withdrawal required hospital admission; 4 patients (50.0%) had documentation of follow-up at 30 days. Adjusted odds ratios for patients who reported fentanyl use compared with patients who reported other opioid use were 0.60 (95% CI, 0.32-1.07) for administered or prescribed buprenorphine in the ED encounter, 1.09 (95% CI, 0.62-1.92) for follow-up at 7 days, and 1.33 (95% CI, 0.73-2.41) for follow-up at 30 days.

**Table 2.** Treatment Characteristics of CA Bridge Study Participants

Characteristic	Participants, No. (%)		
	Fentanyl use		Total (N = 896)
	Yes (n = 87)	No (n = 809)	
Baseline visit reason^a^			
Opioid withdrawal	40 (46.0)	410 (50.7)	450 (50.2)
Recent opioid use or high	9 (10.3)	35 (4.3)	44 (4.9)
Overdose	23 (26.4)	78 (9.6)	101 (11.3)
Seeking medication-assisted treatment	24 (27.6)	226 (27.9)	250 (27.9)
Other opioid use related^b^	10 (11.5)	115 (14.2)	125 (14.0)
Other substance use related	2 (2.3)	29 (3.6)	31 (3.5)
Non–opioid-related reason	4 (4.6)	78 (9.6)	82 (9.2)
Buprenorphine			
Not administered, no prescription	27 (31.0)	231 (28.6)	258 (28.8)
Administered, no prescription	17 (19.5)	125 (15.5)	142 (15.8)
Not administered, prescription	16 (18.4)	130 (16.1)	146 (16.3)
Administered, prescription	27 (31.0)	323 (39.9)	350 (39.1)
First buprenorphine dose^c^			
2-7 mg	5 (11.4)	48 (10.7)	53 (10.8)
8-16 mg	35 (79.5)	383 (85.5)	418 (85)
>16 mg	4 (9.1)	17 (3.8)	21 (4.3)
Total buprenorphine dose^c^			
2-7 mg	4 (9.1)	38 (8.5)	42 (8.5)
8-16 mg	28 (63.6)	363 (81.0)	391 (79.5)
17-24 mg	10 (22.7)	26 (5.8)	36 (7.3)
>24 mg	2 (4.5)	21 (4.7)	23 (4.7)
Buprenorphine prescription dose, median (IQR) [range], mg/d^d^	16 (8-16) [0-32]	16 (8-16) [2-32]	16 (8-16) [0-32]
Buprenorphine prescription days^d^			
1-3	3 (7.0)	28 (6.2)	31 (6.3)
4-7	23 (53.5)	296 (65.6)	319 (64.6)
8-14	10 (23.3)	92 (20.4)	102 (20.6)
>14	7 (16.3)	35 (7.8)	42 (8.5)
Response to administered buprenorphine^c^			
Improved condition	32 (72.7)	325 (72.5)	357 (72.6)
Induced sedation	0	1 (0.2)	1 (0.2)
Adverse events^e^	1 (2.3)	3 (0.7)	4 (0.8)
Precipitated withdrawal	2 (4.5)	6 (1.3)	8 (1.6)
Follow-up treatment engagement^f^			
7-14 d	44 (50.6)	369 (45.6)	413 (46.1)
30 d	36 (41.4)	301 (37.2)	337 (37.6)

^a^ Categories are not mutually exclusive.

^b^ Includes the following categories: abscess, cellulitis, endocarditis, osteomyelitis, seeking opioid pain medication refill, and other.

^c^ The denominator for people who did not use fentanyl is 448. The denominator for people who used fentanyl is 44.

^d^ The denominator for people who did not use fentanyl is 453. The denominator for people who used fentanyl is 43.

^e^ Adverse events include headache, nausea or vomiting, and itchiness.

^f^ Engagement was determined by self-report, electronic health record documentation, or Patient Drug Monitoring Database documentation of an active buprenorphine prescription.

## Discussion

In this cohort study, we observed no differences in follow-up engagement by patients with self-reported fentanyl use (adjusted odds ratio, 1.09), and precipitated withdrawal was rare (8 patients [1.6%]). There are important limitations to our findings; the data were retrospectively collected from the EHR, and both fentanyl use and follow-up engagement were determined through clinical documentation without confirmation and, thus, likely were underreported. Together, these findings show that high-dose buprenorphine administered in the ED for patients in withdrawal is useful in a fentanyl-exposed population.

## Notes

Supplement.
Data Sharing Statement
