# Supplementary material for: High-Dose Buprenorphine Initiation in the Emergency Department Among Patients Using Fentanyl and Other Opioids
Source: JAMA Netw Open. 2023 Mar 3;6(3):e231572. doi: 10.1001/jamanetworkopen.2023.1572 (PMC9984967; doi:10.1001/jamanetworkopen.2023.1572)
Supplement: Supplement. — Data Sharing Statement [file jamanetwopen-e231572-s001.pdf]

Data Sharing Statement

Snyder. High-Dose Buprenorphine Initiation in the Emergency Department Among Patients Using Fentanyl and Other Opioids. *JAMA Netw Open*. Published March 03, 2023. doi:10.1001/jamanetworkopen.2023.1572

Data
Data available: Yes
Data types: Deidentified participant data, Data dictionary
How to access data: An individual interested in accessing de-identified data and the data dictionary will contact Dr. Hannah Snyder to request access to the dataset. The request to share data can be made by providing a detailed plan from the investigator asking for the dataset to describe the concept of interest, to provide a brief outline of a timeline for analyzing the data and preparing a manuscript, to submit a copy of the curriculum vitae of the investigator making the request and to provide documentation of proof of training in the ethical treatment of human subjects in research. The form will be submitted directly to Dr. Hannah Snyder at hannah.snyder@ucsf.edu
When available: With publication

Supporting Documents
Document types: None

Additional Information
Who can access the data: Research whos use has been approved as above.
Types of analyses: Any purpose
Mechanisms of data availability: After approval as above and with a signed agreement
